# Time course of focused ultrasound effects on β-amyloid plaque pathology in the TgCRND8 mouse model of Alzheimer’s disease

**DOI:** 10.1038/s41598-018-32250-3

**Published:** 2018-09-19

**Authors:** Charissa T. Poon, Kairavi Shah, Chiungting Lin, Ryan Tse, Kate K. Kim, Skyler Mooney, Isabelle Aubert, Bojana Stefanovic, Kullervo Hynynen

**Affiliations:** 1 Physical Sciences Platform, Sunnybrook Research Institute, Toronto, Ontario Canada; 2 Institute of Biomaterials and Biomedical Engineering, University of Toronto, Toronto, Ontario Canada; 3 Department of Medical Biophysics, University of Toronto, Toronto, Ontario Canada; 4 Biological Sciences Platform, Sunnybrook Research Institute, Toronto, Ontario Canada; 5 Department of Laboratory Medicine and Pathobiology, University of Toronto, Toronto, Ontario Canada

## Abstract

Previous studies have demonstrated that temporarily increasing the permeability of the blood-brain barrier using focused ultrasound can reduce β-amyloid plaque load and improve cognitive function in animal models of Alzheimer’s disease. However, the underlying mechanism and duration for which the effects of one treatment persists for are unknown. Here, we used *in vivo* two-photon fluorescence microscopy to track changes in β-amyloid plaque sizes in the TgCRND8 mouse model of Alzheimer’s disease after one focused ultrasound treatment. We found that one treatment reduced plaques to 62 ± 16% (*p* ≤ 0.001) of their original volume two days post-sonication; this decrease in size persisted for two weeks. We then sought to evaluate the effectiveness of biweekly focused ultrasound treatments using magnetic resonance imaging-guided focused ultrasound treatments. Three to five biweekly treatments resulted in a 27 ± 7% (*p* ≤ 0.01) decrease in plaque number and 40 ± 10% (*p* ≤ 0.01) decrease in plaque surface area compared to untreated littermates. This study demonstrates that one focused ultrasound treatment reduces the size of existing β-amyloid plaques for two weeks, and that repeated biweekly focused ultrasound treatments is an effective method of reducing β-amyloid pathology in moderate-to-late stages of Alzheimer’s disease.

## Introduction

Despite the rising prevalence of Alzheimer’s disease (AD)^1^, there are currently no disease-modifying drugs for the treatment of AD. Hallmarks of AD include accumulation of extracellular β-amyloid (Aβ) peptides, intracellular aggregations of hyperphosphorylated tau, neuronal degeneration, and loss of cognitive and memory functions. Part of the challenge in treating neurological diseases such as AD is the presence of the blood-brain barrier (BBB), which limits almost all large-molecule therapeutics and more than 98% of small-molecule drugs from entering the brain parenchyma^2^. Proposed mechanisms to bypass the BBB are invasive, and produce global, heterogeneous, and unpredictable effects in the brain^3,4^.

One potential solution to circumventing the BBB is to use focused ultrasound (FUS). FUS, used in conjunction with intravenously injected microbubbles, can transiently and safely increase the permeability of the BBB^5–8^. Microbubbles expand and contract in the focus of the propagating ultrasound beam, thereby increasing transcellular and paracellular transport^9,10^. This increase in BBB permeability persists for approximately 10 to 24 h^5,11^. Various compounds, including stem cells^12^, chemotherapeutics^13^, and viral vectors^14^ have been delivered to select brain regions using FUS.

The use of FUS for the treatment of AD has been previously assessed. In 2010, Jordão *et al*. reported that unilateral delivery of an anti-Aβ antibody using FUS led to a significant reduction in Aβ plaque load, specifically plaque number and size, in the treated compared to the untreated cortex of TgCRND8 mice, which present an aggressive form of AD^15^. It was discovered that one treatment of FUS itself, without therapeutics, also significantly decreased Aβ plaque load^16^. Since repeated FUS treatments would likely be necessary for neurodegenerative diseases like AD, Burgess *et al*. assessed the effect of administering three weekly treatments targeted to the bilateral hippocampi in TgCRND8 mice^17^. Compared to untreated mice, FUS-treated transgenic mice exhibited reduced hippocampal Aβ plaque load, improved performance in the novel arm Y-maze test, and increased doublecortin immunopositive immature neurons in the dentate gyrus^17^. The effects of weekly FUS treatments in reducing Aβ plaque load and extracellular Aβ species were confirmed in the APP23 amyloidogenic^18^ and pR5^19^ tau mouse models of AD, respectively. Using a cholesterol-induced AD rabbit model, Alecou *et al*. also found that three FUS treatments, delivered three days apart, reduced the number of Aβ plaques compared to untreated rabbits^20^.

There are two unknown factors in these studies. First, it is unclear how FUS reduces Aβ plaque load. While previous trials have demonstrated that FUS leads to a decrease in Aβ plaque number, size, and brain surface area covered by Aβ compared to untreated brain regions^15,16^ or animals^17,18^, it is unknown if FUS causes a decrease in the size of existing plaques, or impedes the growth of existing plaques. Understanding how FUS affects amyloid pathology will be useful in selecting the most effective pharmacological agents to be used in conjunction with FUS treatments. For example, BACE1 inhibitors reduces the *de novo* generation of Aβ^21^, whereas anti-Aβ antibodies can increase amyloid phagocytosis^22^ or prevent Aβ seeding or aggregation^23^.

Second, while repeated FUS treatments separated by intervals of three days^20^ and one week^17,18^ proved to be effective in reducing Aβ load in FUS-treated compared to untreated controls, the optimal treatment interval is currently unknown. It is therefore necessary to investigate how the effects of FUS treatments on AD pathology changes with time, and whether repeated treatments have an additive effect.

**Figure 1:**
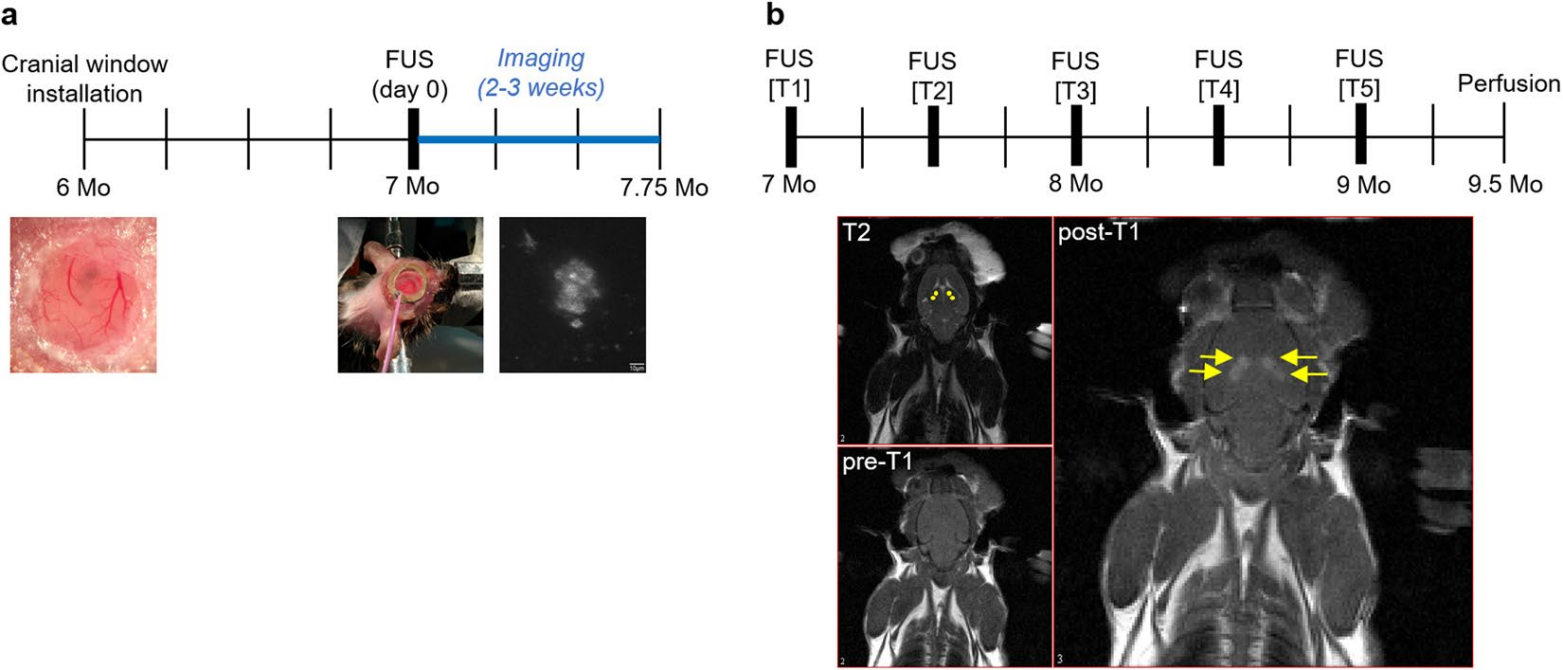
Timelines of two-photon fluorescence microscopy and MR-guided FUS experiments. Two-photon fluorescence microscopy FUS experiments. (**a**) Cranial windows were installed in the parietal bone when mice were 6-months-old. Mice were given three to four weeks to recover to allow surgery-related inflammation to recede. On day 0 of imaging, Tg FUS animals were sonicated immediately after administration of microbubbles, whereas the transducer was disconnected during sonication for Tg CTL animals. Following sonication, XYZ image stacks of up to ten plaques per animal were collected. Subsequent imaging sessions were on days 2, 4, 7, 10, 12, 14, and 21. Each vertical line indicates a week. MR-guided FUS experiments. (**b**) *Top:* Mice were administered five FUS treatments biweekly, over a total of 10 weeks. Subjects were entered into the study at 7-months-old, and sacrificed at 9.5-months-old. Each vertical line indicates a week. *Bottom*: For each FUS treatment, three MRIs were taken. A T2-weighted MRI was taken to allow anatomical targeting of the bilateral hippocampi. Two targets were chosen for each hippocampus (T2, yellow dots). A baseline T1-weighted image was taken before FUS treatment (pre-T1). Immediately after FUS treatment, gadolinium contrast agent was injected intravenously, and a post-sonication T1-weighted image was taken to confirm BBB opening at the targeted locations (post-T1, yellow arrows). Mo = months.

Here, we address these questions through two sets of experiments (Fig. 1) in 7-month-old TgCRND8 mice, an age that reflects moderate-to-severe stage of AD^24^. First, to measure the effectiveness of one FUS treatment in AD and to discern if FUS causes a decrease in the size of existing Aβ plaques, plaques in FUS-treated and untreated TgCRND8 animals were tracked using two-photon fluorescence microscopy. Two-photon fluorescence microscopy allows *in vivo* imaging of the brain through a cranial window, thus providing a way to follow changes in Aβ plaque size after FUS. By tracking plaques in FUS-treated and untreated TgCNRD8 animals over a maximum of three weeks, we demonstrated that one FUS treatment causes a significant decrease in plaque size from two days to two weeks after FUS treatment. Therefore, in the second experiment, TgCRND8 animals were treated with magnetic resonance imaging-guided FUS (MRgFUS) treatments targeted to the bilateral hippocampi once every other week, for a total of ten weeks (five FUS treatments total). Immunohistochemical analysis of Aβ plaque number, maximum cross-sectional area, and surface area covered by Aβ plaques revealed that biweekly treatments were effective in reducing the severity of Aβ pathology in brain tissue that was extensively covered by Aβ plaques.

## Results

### Two-photon fluorescence microscopy FUS experiments: Plaque tracking

#### FUS-mediated increase in BBB permeability was confirmed by leakage of fluorescent dextran from the intravascular to extravascular space

**Figure 2:**
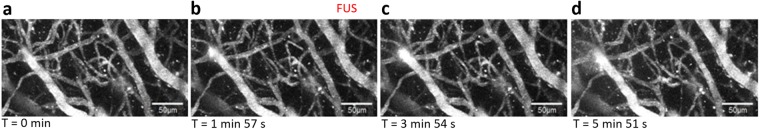
Confirmation of FUS treatment in two-photon microscopy experiments. Maximum projection images of XYZ image stacks are shown. FUS-mediated increases in BBB permeability can be observed by the leakage of fluorescently conjugated dextran from the blood vessels into the extravascular space (**b–d**). FUS was administered in panel *b*. Scale bar = 50 μm.

Prior to sonication, arterioles, venules, and capillaries were visible from intravenous administration of Texas Red 70 kDa (TR70) conjugated dextran. During and after sonication, enhanced BBB permeability was confirmed by the leakage of dextran from the intravascular into the extravascular space (Fig. 2).

#### Aβ plaques were found by localizing them in relation to neighbouring blood vessels

A ‘plaque map’ was created on the first day of imaging by stitching together XYZ-stacks of plaques and surrounding amyloid-covered blood vasculature (Supplementary Fig. S1). From this, each plaque’s location was mapped to nearby amyloid-covered vessels, which facilitated identification of the same plaques over different imaging days (Supplementary Fig. S2). A small number of Aβ plaques were found to move relative to blood vessels. Image stacks of plaques were discarded if there were breathing artifacts or if signal-to-noise (SNR) ratio was poor. A total of 52 plaques were imaged in TgCRND8 animals for a maximum of 21 days.

#### Aβ plaques in Tg CTL animals increased significantly in volume by day 14, and in cross-sectional area by day 21

**Figure 3:**
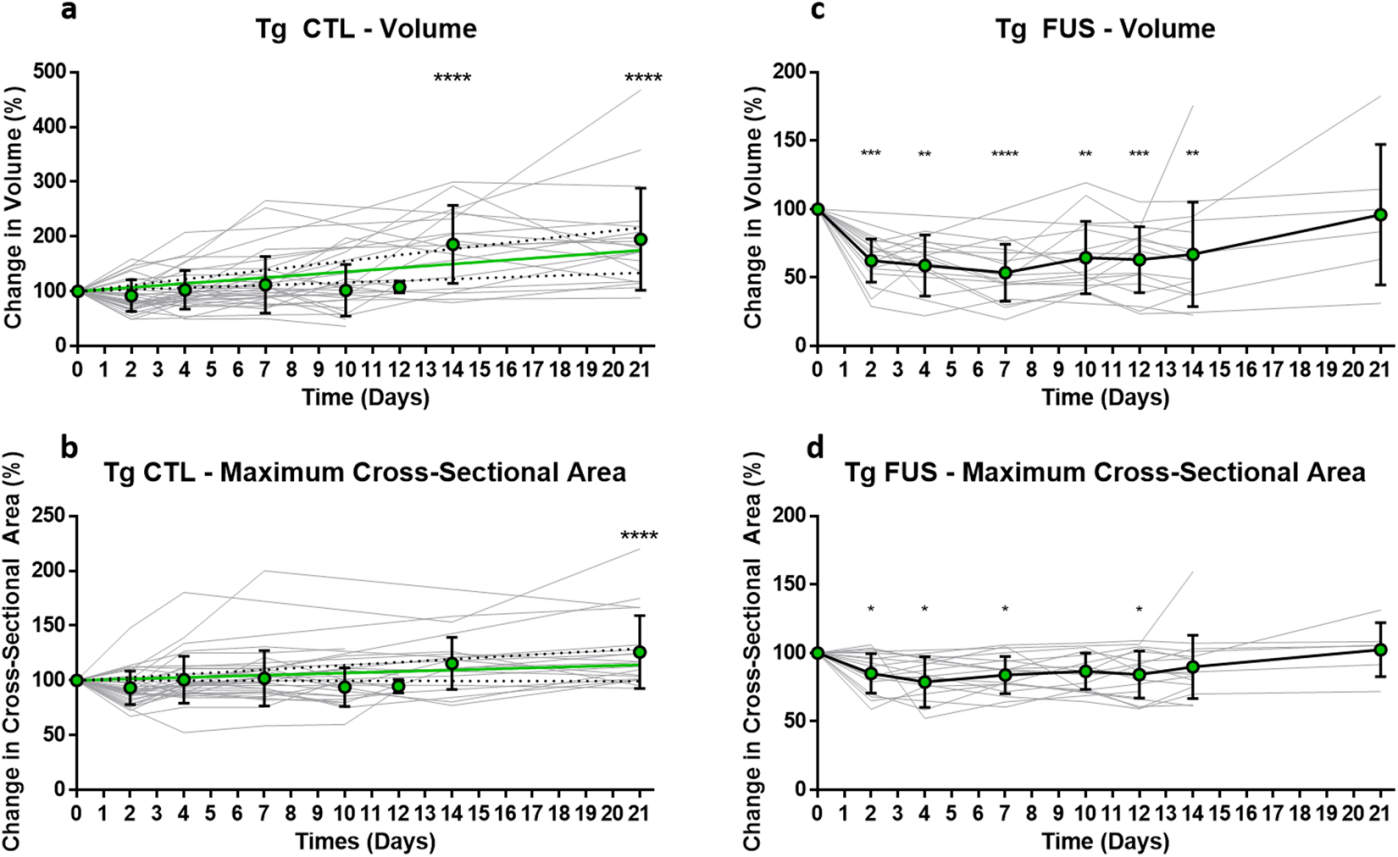
Changes in plaque volume in Tg CTL and Tg FUS animals over three weeks. Plaque volumes of 7-month-old Tg mice were assessed by taking depth-stacks on day 0, 2, 4, 7, 10, 12, 14, and 21, and normalizing them to the volume obtained on day 0, where day 0 is the first day of imaging. Solid gray lines represent individual plaques, green dots and error bars show mean and SD values. Tg CTL animals demonstrated a linear increase in plaque growth over three weeks. (**a**,**b**) Tg CTL animals showed a linear increase in plaque volume (3.6 ± 0.8% per day, 95% CI slope = 1.6 to 5.5, Sy.x = 25.6), reached significance on day 14 (186 ± 71%) and peaked on day 21 (195 ± 93%). (**a**) Similarly, Tg CTL animals showed a linear increase in plaque cross-sectional area (0.7 ± 0.3% per day, 95% CI slope = 0.0 to 1.4, Sy.x = 9.2), although significance was only achieved on day 21 (126 ± 33%). The solid green line represents the linear regression, and the dotted black lines show the 95% CI of the linear regression. (**b**) Tg FUS animals demonstrated a rapid decrease in plaque volume by day 2 after FUS treatment. (**c**,**d**) Tg FUS animals showed a sharp and significant decrease in plaque volume by day 2 (62 ± 16%); this significant difference was maintained until day 14 (67 ± 38%). By day 21, plaque volume returned to the starting value measured on day 0 (96 ± 51%). (**c**) Similarly, Tg FUS animals showed a rapid and significant decrease in plaque cross-sectional area by day 2 (85 ± 15%); this significance was also maintained until day 7, and returned to baseline levels by day 14 (90 ± 23%). (**d**) Data tables can be found in Supplementary Tables S11–S12. *p ≤ 0.05, **p ≤ 0.01, ***p ≤ 0.001, ****p ≤ 0.0001, *n* = 4 for Tg CTL, *n* = 5 for Tg FUS.

As a positive control, Aβ plaques in transgenic control (Tg CTL) animals were tracked and measured. Thirty-six plaques were evaluated in Tg CTL animals (*n* = 4, Fig. 3a,b). To find differences in plaque volume and maximum cross-sectional area over time, measurements were normalized to the volume and cross-sectional area values obtained on the first day of imaging (day 0). The mean and standard deviation of these measurements were then calculated and plotted. A linear regression of plaque volume and cross-sectional area measurements revealed a linear growth rate (volume: 3.6 ± 0.8% per day, 95% CI slope = 1.6 to 5.5, Sy.x = 25.6; cross-sectional area: 0.7 ± 0.3% per day, 95% CI slope = 0.0 to 1.4, Sy.x = 9.2). Two and three weeks after the onset of imaging, plaque volumes increased significantly compared to that measured on day 0 (two weeks: 186 ± 71%, *p* ≤ 0.0001; three weeks: 195 ± 93%, *p* ≤ 0.0001). The maximum cross-sectional area of plaques did not increase at the two-week time point (115 ± 24%, *p* = 0.13), but did at the three-week time point (126 ± 33%, *p* ≤ 0.0001). Larger and smaller plaques showed similar rates of plaque growth (Supplementary Fig. S3).

#### One FUS treatment significantly reduced Aβ plaque volume at two days post-sonication; decreased plaque volumes persisted for two weeks

Laser powers used on day 0 were found to be consistently higher than those used on subsequent imaging days in FUS-treated animals due to the leakage of fluorescent dextran into the extravascular space during FUS-induced BBB treatment. Since image stacks of plaques were acquired after FUS treatment, signal from the dextran channel (TR70) bled into the plaque channel (methoxy-X04), increasing background fluorescence, and thereby requiring higher laser power to achieve adequate SNR. Notably, laser powers used on day 0 were consistent with all other imaging days in CTL animals due to the lack of FUS exposure. In addition, increases in laser power correlated with increases in measured plaque volume and maximum cross-sectional area. Thus, we performed the same plaque imaging at a range of laser powers to develop a correction factor that was applied to plaque volume and maximum cross-sectional area measurements obtained on day 0 (*Supplementary Methods*).

Eighteen plaques were tracked and measured in transgenic FUS (Tg FUS) animals (*n* = 5, Fig. 3c,d). The same analyses as those detailed above for Tg CTL animals were used. Two days after FUS treatment, Aβ plaques decreased significantly in volume compared to their starting volume (62 ± 16%, *p* ≤ 0.001). This difference persisted until day 14 (67 ± 38%, *p* ≤ 0.01), but increased by day 21 (96 ± 51%). Similarly, a significant decrease in maximum cross-sectional area was observed as early as day 2 (85 ± 15%, *p* ≤ 0.05), which persisted until day 7 (84 ± 14%, *p* ≤ 0.05), but increased by day 14 (90 ± 23%). Larger and smaller plaques showed similar rates of changes in plaque volume and maximum cross-sectional area (Supplementary Fig. S3). Linear regression from day 2 to day 21 revealed a slower increase in plaque volume but not maximum cross-sectional area in Tg FUS compared to Tg CTL animals (volume: 1.7 ± 0.5% per day, 95% CI slope = 0.4 to 3.1, Sy.x = 8.5; cross-sectional area: 1.0 ± 0.3% per day, 95% CI slope = 0.3 to 1.7, Sy.x = 4.1).

### MR-guided FUS: Five weekly FUS treatments

#### Multiple FUS treatments were well-tolerated in old TgCRND8 mice

Forty-two animals were entered into this study. Of the 20 Tg animals, 13 were designated into the Tg FUS group, and seven into the Tg CTL group. The uneven group sizes were due to concerns of old TgCRND animals’ tolerance of repeated treatments. Of the 22 nTg animals, 11 were designated into the nTg FUS group, and 11 into the nTg CTL group. Throughout the ten weeks of the study, two Tg FUS, one Tg CTL, three nTg FUS, and one nTg CTL mice died. There was no significant difference in mortality between genotypes (Tg vs nTg, p > 0.99) or treatment (FUS vs CTL, p = 0.68, Supplementary Fig. S4). Of the remaining animals, only those who received at least three treatments over ten weeks were included in the immunohistochemical analysis (Supplementary Table S5). The primary reason for a missed treatment was the challenge of inserting a tail vein catheter, which was a concern in the latter treatments.

#### Mice in all four groups maintained similar weights throughout the study

Weight was one of the assessments of animal health in this study (Supplementary Fig. S6). All subjects’ weights fluctuated within 13% of their starting weight measured before treatment 1. A two-way ANOVA revealed that the main effect of genotype on weight fluctuations was significant (*p* = 0.0002), such that Tg animals exhibited more weight fluctuations than nTg animals. In contrast, the main effect of treatment number on weight fluctuations was not significant (*p* > 0.9).

#### FUS-mediated increases in BBB permeability as measured by MR enhancement images were not significantly different between Tg and nTg animals

Increases in BBB permeability were evaluated by comparing the pixel enhancement values of FUS-targeted regions with untargeted regions in the brain, in T1-weighted MRIs. Tg and nTg animals showed an average relative enhancement value of 146 ± 23% and 153 ± 24% compared to untreated brain regions, respectively (mean ± SD, *n* = 5 per group, Supplementary Fig. S7). A *t*-test indicated that the difference was not significant.

#### Plaque number and surface area in the hippocampus significantly decreased with three to five biweekly FUS treatments

**Figure 4:**
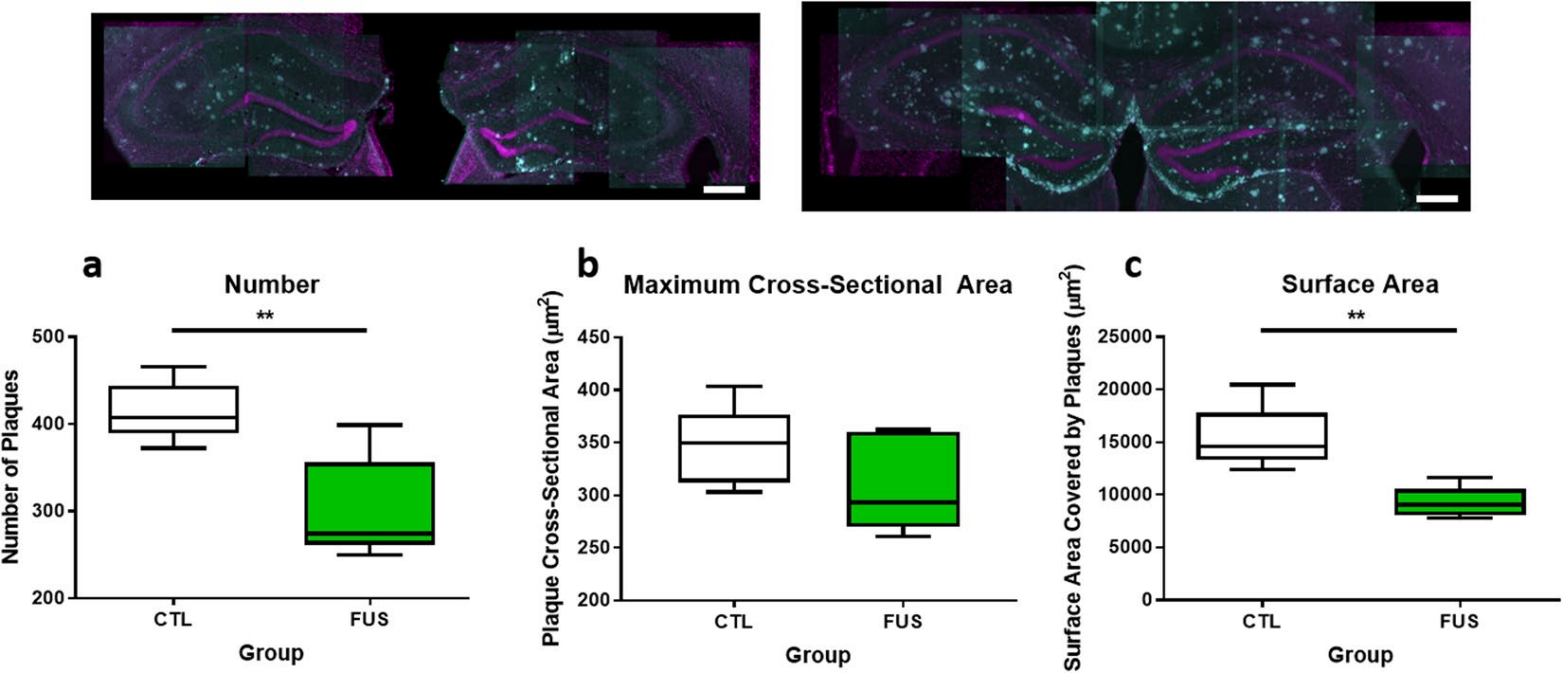
Boxplots showing comparisons of plaque number and surface area between Tg FUS and Tg CTL animals. *Top*: Representative maximum projection images of hippocampi in Tg FUS (left) and Tg CTL (right) animals (magenta = NeuN, cyan = 6F3D). *Bottom:* Plaque number (**a**) in Tg FUS mice decreased significantly by 27 ± 7% compared to Tg CTL littermates (300 ± 30 and 420 ± 20, respectively, *p* = 0.006). Maximum cross-sectional area of plaques (**b**) in Tg FUS mice was comparable to that observed in Tg CTL littermates (350 ± 20 and 310 ± 20 µm^2^, respectively, *p* = 0.24). Plaque surface area (**c**) in Tg FUS mice decreased significantly by 40 ± 10% compared to Tg CTL littermates (9270 ± 660 and 15400 ± 1360 µm^2^, respectively, *p* = 0.004). Data tables can be found in Supplementary Table S13. Error bars show mean ± SD, *n* = 5 for each group, **p ≤ 0.01.

After three to five biweekly treatments, Aβ plaque number, maximum cross-sectional area, and surface area were evaluated in the hippocampi of Tg mice (Fig. 4). The mean number of plaques decreased significantly by 27 ± 7% (*p* ≤ 0.01) between Tg FUS (300 ± 30) and Tg CTL subjects (420 ± 20). Maximum cross-sectional area was unchanged between Tg FUS (310 ± 20 μm^2^) and Tg CTL subjects (350 ± 20 μm^2^; difference between means of 10 ± 8%, *p* = 0.24). Plaque surface area also decreased significantly by 40 ± 10% (*p* ≤ 0.01) between Tg FUS (9270 ± 660 μm^2^) and Tg CTL subjects (15350 ± 1360 μm^2^) (mean ± SD, *n* = 5 for each group).

## Discussion

To our knowledge, this is the first study that characterizes the temporal efficiency of single FUS treatments in AD animals. Specifically, we report that one FUS treatment reduces the size of existing Aβ plaques at two days to two weeks after sonication, and demonstrate that repeated treatments administered in two-week intervals have an additive effect in reducing Aβ pathology despite the progression of amyloid pathology in animals with moderate-to-severe AD.

We used two-photon fluorescence microscopy, which has exceptional depth penetration and optical sectioning^25^, to image Aβ plaques at high spatial resolution over the course of weeks. Using methoxy-X04, which exhibits excellent *in vitro* binding affinity for Aβ_1–40_ fibrils (K_i_ = 26.8 nM) and shows equivalent *in vivo* labelling specificity as thioflavin-S^26^, we provide evidence that Aβ plaques were significantly reduced in size as early as  two days, and up to 14 days, after one FUS treatment. Thus, the difference in Aβ plaque load observed between FUS-treated and untreated littermates in previous studies is likely due to an active reduction in the size and number of existing Aβ plaques. While decreases in Aβ plaque load may also be caused by a suppression of Aβ aggregation, the effect of FUS on Aβ aggregation was not addressed in this study.

Since one FUS treatment has a therapeutic time window of at least two weeks, we then asked if multiple FUS treatments given once every two weeks have an additive effect on reducing Aβ pathology. Analysis of Aβ plaques in MRgFUS-treated mice was conducted using 6F3D antibody, which is specifically directed against Aβ_8–17_^27^. Our results show that biweekly treatments are as effective as weekly treatments in improving Aβ pathology. Specifically, biweekly treatments in 9.5-month-old TgCRND8 mice resulted in a 27 ± 7% (*p* = 0.006) decrease in plaque number, 10 ± 8% (*p* = 0.24) decrease in maximum cross-sectional area, and 40 ± 10% (*p* = 0.004) decrease in surface area. In contrast, three weekly treatments resulted in a 19% decrease in plaque number and 20% decrease in plaque size in 8-month-old TgCRND8 mice^17^. In addition, many subjects in this study did not receive all five FUS treatments due to the challenge of inserting of tail vein catheters repeatedly (Supplementary Table S5); thus, our results likely reflect a diluted effect size. The 1.5-month difference in subjects’ ages in the two studies is also notable due to the aggressive acceleration of plaque pathology observed in the TgCRND8 mouse model^24,28^, which has been correlated with impairments in memory processes^29^. The significant reductions in plaque number and surface area after repeated biweekly treatments suggests that FUS, delivered at two-week intervals, has an additive effect in reducing Aβ plaque pathology in animals with moderate to severe AD.

Previous studies have examined possible mechanisms of FUS-mediated reductions of Aβ plaques. Binding of endogenous immunoglobulins to Aβ plaques has been observed in FUS-treated AD mice at 4 h and 4 days post-sonication^16^. In addition, FUS induced activation and increased phagocytosis of Aβ in microglia and astrocytes^16^, particularly in microglial lysosomal compartments^18^. Since microglia are highly motile cells and the primary phagocytic cell type in the CNS, it is likely that they contributed to the rapid decrease in Aβ plaque size that was observed 48 h after sonication in this study. In addition, it is plausible that FUS induces the infiltration of systemic phagocytic immune cells^30^ into the brain, which can aid in Aβ plaque clearance. Recent studies have shown that FUS results in an elevation of monocyte chemotactic protein-1 (MCP-1) and migration of CD68+ systemic macrophages into the brain, although the microbubble concentration used in these studies likely caused damage^31–33^.

An acute inflammatory response, including upregulation of proinflammatory cytokine genes, has been observed post-sonication, although much of this response returns to baseline by 24 h^32^. Some proinflammatory cytokines, such as IL-1β and IL-6, have been associated with reduced amyloidosis and amelioration of cognitive deficits in APP mouse models^34,35^, and may contribute to the beneficial effects of FUS in AD. Our results indicate that FUS-mediated Aβ clearance mechanisms persist up to 14 days after sonication. Future FUS studies should include longer time points to evaluate long-term effects.

Various degrees of BBB dysfunction have been reported in human AD^36–39^. In contrast to other studies^40,41^, we did not detect any qualitative differences in basal BBB permeability between Tg and nTg animals in our MRgFUS nor two-photon microscopy studies. FUS-mediated BBB effects were evident from the leakage of gadolinium contrast agent exclusively in the targeted areas (Fig. 1). Although there are concerns that FUS may increase the prevalence of cerebral microbleeds in AD^42–44^, it is notable that as hemosiderin deposits and extravasated erythrocytes are rarely observed after FUS treatments^7^. Thus, the effects of FUS on BBB permeability appear to be distinct from the vascular pathologies observed in AD. Notably, no significant changes in the characteristics of FUS-mediated increases in BBB permeability were observed between Tg and nTg mice^17,45,46^.

Characteristics of increasing BBB permeability can vary as a function of FUS parameters, microbubble activity^47^, species, and brain region^45^. However, safe FUS treatments can be consistently delivered by using an acoustic controller^17,48^. An acoustic controller increases pressures until microbubble emissions that are characteristic of stable cavitation are detected, thereby increasing BBB permeability and remaining below the threshold for tissue damage^48,49^. Using this method, successful BBB opening has been achieved in various animal models^17,48,50^ without causing edema, vacuolations, or neuronal loss, and with minimal to no erythrocyte extravasation^48^.

Open-skull cranial windows have been associated with increased gliosis and absence of robust plaque growth^51,52^. However, plaques in Tg CTL animals exhibited significant increases in size during the three weeks of imaging, and plaques in Tg FUS animals also increased in size by the three-week time point, demonstrating that cranial windows did not prevent Aβ plaque growth in this study. It has also been shown that different dosing regimens of methoxy-X04 do not result in differences in plaque growth evaluated by two-photon fluorescence microscopy^53^; therefore, it is unlikely that the observed plaque growth is due to accumulated methoxy-X04. Decreases in Aβ size observed in Tg FUS but not Tg CTL animals also suggested that changes in plaque size were not caused by the imaging itself.

One limitation to our study is that the laser powers used on day 0 in FUS-treated animals were consistently higher than all other imaging days, due to the leakage of dextran into the extravascular space, thereby requiring higher laser powers to achieve adequate SNR during image acquisition of plaques. A correction factor was therefore developed by finding the relationship between laser power and measured plaque volume and maximum cross-sectional area (Supplementary Methods). Another limitation is the variance in rate of plaque growth, although this is expected due to varied proximity to the sites of BBB opening, varied initial plaque sizes, and complexity of AD.

Therapies that can be administered repeatedly are key in treating chronic diseases like AD. Repeated FUS treatments covering a large brain volume have been shown to be well-tolerated in non-human primates^7,8^. The effects of FUS on reducing Aβ^16–18,20^ and tau^19^ pathology, ameliorating memory deficits^17,18^, and increasing neurogenesis^17,54^ are promising in treating AD. Future work should focus on interrogating the biophysical mechanisms driving FUS amelioration of AD pathogenesis, and on investigating pharmacological agents that would be most effective when used in conjunction with FUS.

## Materials and Methods

### Experimental design

All analyses were conducted blinded to the genotype and treatment group. Randomization was performed by choosing groups based on position of animal cages on the racks. A table of the two experiments can be found in Supplementary Table S8.

### Animal preparation for all FUS experiments

All procedures were approved by Sunnybrook Research Institute’s Animal Care and Use Committee and conducted in accordance with the guidelines set by the Canadian Council on Animal Care. Since Aβ levels do not differ between male and female TgCRND8 mice^24^, both sexes were used in this study. TgCRND8 mice were used due to the aggressive progression of Ab pathology in this model.

For two-photon fluorescence microscopy experiments, a total of nine female 6-month-old TgCRND8 mice were split into FUS-treated (‘Tg FUS’, *n* = 5) and control (‘Tg CTL’, *n* = 4) groups. A separate cohort of 42 male and female 7-month-old TgCRND8 mice were used for MRgFUS experiments. Subjects were split into transgenic FUS-treated (‘Tg FUS’, *n* = 13), transgenic control (‘Tg CTL’, *n* = 7), non-transgenic FUS-treated (‘nTg FUS’, *n* = 11), and non-transgenic control (‘nTg CTL’, *n* = 11) groups. Transgenic animals were preferentially separated into the Tg FUS group in anticipation of experimental difficulties. Timelines for MRgFUS and two-photon fluorescence microscopy experiments are shown in Fig. 1.

Mice were taken out of the study and sacrificed if they met any of the following endpoints: failure to groom, weight loss exceeding 20% of normal body mass, abdominal distention, persistent self-trauma, abnormal locomotion, vocalization, abnormal discharge.

### Two-photon fluorescence microscopy FUS experiments

#### Animal preparation for two-photon fluorescence microscopy experiments

Chronic cranial windows were installed when animals were 6-months-old such that they would be 7-months-old after three to four weeks of recovery^55^. The procedure for creating semi-sterile cranial windows in mice is well described in literature^53,55,56^. Briefly, animals were anesthetized with 2% isoflurane in a mix of medical air and oxygen. Body temperature was maintained at 37 °C using a rectal probe and heating pad fixed to the stereotaxic frame. All surgical tools, supplies, and drapes were autoclaved within 48 h of surgery. Prior to surgery, animals were administered dexamethasone sodium phosphate (0.2 mg/kg, intramuscular injection) and carprofen (5 mg/kg, intraperitoneal injection) to reduce edema and inflammation, respectively. Fur in the surgical area was removed with depilatory cream, and then cleaned with three alternating swabs of iodopovidone and alcohol. The scalp directly above the skull was removed and the periosteum was pushed back from the skull surface. A circular piece of skull 3–4 mm in diameter was removed from the parietal bone using a dental drill. The dura was left intact. The exposed brain was covered with a circular coverslip (5 mm diameter, # 1 thickness, Warner Instruments, Connecticut, USA), and secured to the skull using cyanoacrylate glue. Animals were recovered under a heat lamp, and given daily doses of carprofen (5 mg/kg), ketoprofen (5 mg/kg), antibiotics (Baytril, Bayer Corps, Kansas, USA), polysporin, and soft foods for three days post-surgery. Animals were given three to four weeks to recover prior to the onset of imaging to allow for the window to clear and inflammation to resolve^52,55^.

#### Two-photon fluorescence microscopy

All imaging was done with the FV1000MPE multiphoton laser scanning microscope (Olympus, Tokyo, Japan) and Ti:Sa laser (MaiTai, Spectra-Physics, Darmstadt, Germany).

To visualize dense-core plaques, methoxy-X04 (Tocris, Bio-Techne Corporation, Minneapolis, USA) was injected intraperitoneally 24 h prior to each imaging session (diluted in 10% DMSO, 45% propylene glycol, 45% saline; delivered 5 mg/kg)^26^. To visualize blood vessels, 70 kDa Texas Red dextran (Invitrogen, Burlington, Canada; dissolved in PBS; 5 mg/kg) was injected intravenously through a tail vein. Two excitation wavelengths were used: 750 nm for methoxy-X04^26^, and 900 nm for Texas Red (Tg FUS: 14.1 ± 11.9 mW, Tg CTL: 6.81 ± 3.29 mW, mean ± SD; see *Supplementary Methods* for all laser powers used). Texas Red was only injected on the first day of imaging (day 0).

On the first day of imaging (day 0), a water-immersion objective with 40× magnification power was used (LUMPLFLN 40×, NA: 0.80, Olympus, Tokyo, Japan). The objective lens was aligned with the center of the ring transducer. To observe blood vessel dynamics during sonication, XYZT stacks were collected. The following imaging parameters were used: 512 × 512 pixels (XY), 0.310 μm/pixel, 5 μm step-size for 250–300 μm total (Z), 12.5 μs/pixel.

On subsequent experiment days, a water-immersion objective with 25× magnification power was used (XLPLN 25×, NA: 1.05; Olympus, Tokyo, Japan). Only images of plaques were collected. XYZ-stacks of plaques were acquired at a higher zoom: 512 × 512 pixels (XY), 0.310 μm/pixel or 0.331 μm/pixel, 2 μm step-size for 40–200 μm total (Z), 12.5 μs/pixel.

An average of ten plaques were imaged per animal. XYZ-stacks of every plaque were obtained on day 0 (FUS treatment) and subsequently on days 2, 4, 7, 10, 12, 14, and 21. Tg FUS mice were only given FUS treatment once (day 0).

#### Focused ultrasound treatments in two-photon microscopy experiments

For FUS treatments, a PZT-4 cylindrical transducer (10 mm diameter, 1.5 mm thickness, 1.1 mm height) was driven at 1.1 MHz in thickness mode, producing a circular focal spot^57^. The depth of field of this transducer is 1 mm beneath the coverslip. The transducer was controlled by a function generator (Agilent, Palo Alto, CA, USA) and a 53 dB RF power amplifier (NP Technologies, Inc., Newbury Park, CA, USA); forward and reflected RF powers were measured using a power meter built in-house (Supplementary Fig. S9).

On the first day of imaging (day 0), the ring transducer was mounted onto the cranial window. Both Tg FUS and Tg CTL animals were given an injection of Definity microbubble (MB) contrast agent (Lantheus Medical Imaging, North Billerica, MA), diluted 1:10 in saline (v/v), and delivered at a dose of 0.04 mL/kg^46^. Immediately after MB injection, Tg FUS animals were sonicated using the following FUS parameters: 10 ms pulse duration, 1 Hz pulse repetition frequency, 120 s total sonication duration, with estimated *in situ* pressures of 0.4–0.8 MPa^46,58,59^. The transducer was disconnected from the amplifier for Tg CTL animals. Successful FUS treatment was determined by the leakage of fluorescent dextran from blood vessels into the extravascular space, indicating increased BBB permeability.

#### Image processing

Two-photon fluorescence images were analyzed using a MATLAB script (MATLAB and Statistics Toolbox Release 2015, The MathWorks, Inc., Natick, MA, USA) written in-house (script available upon request). User input was limited to selecting the ROI containing the plaque in each image stack, thereby minimizing user bias. Our image processing algorithm involved five main steps: (1) importing image stack, (2) identifying ROI, (3) iterative thresholding^60^, (4) defragmenting, (5) computing volume (Supplementary Fig. S10). The only step that required user input was the selection of a ROI containing the plaque. In the thresholding step, we used an iterative thresholding process^60^ to binarize the image stack into ‘foreground’ and ‘background’ pixels. In the defragmenting step, the foreground was filtered to isolate the pixels that were part of the plaque by evaluating if a sufficient number of neighbouring pixels were also ‘foreground’ pixels. Finally, the plaque volume and maximum cross-sectional area were calculated by counting the number of filled pixels surrounding the seed pixel that shared a face with a counted pixel (Supplementary Fig. S10).

To determine if the rate of change in plaque size differentially affected larger or smaller plaques, plaques were binned as ‘larger’ or ‘smaller’ based on the median plaque volume in each dataset.

#### Statistical analysis

Statistical analyses were performed using GraphPad Prism (Prism version 7.03 for Windows, GraphPad Software, La Jolla California USA). To evaluate changes in plaque volume and maximum cross-sectional area, measurements obtained on days 2, 4, 7, 10, 12, 14, and 21 were normalized to measurements obtained on day 0 and expressed as a percentage. A one-way ANOVA and Holm-Sidak multiple comparisons test was used to compare differences in plaque size at every imaging day relative to that on day 0 (α = 0.05). A t-test was used to compare rates of change in plaque size between larger and smaller plaques.

### MR-guided FUS experiments

#### Animal preparation for MR-guided FUS experiments

Prior to every FUS treatment, mice were anesthetized using 5% isoflurane in medical air^61–63^. Once a sufficient plane of anesthesia was achieved, mice were weighed and maintained at 2% isoflurane. A 27-gauge catheter was inserted into a tail vein for intravenous access. Since air bubbles in the fur will obstruct the propagation of ultrasound, all fur on the head was removed using depilatory cream.

#### Magnetic resonance-guided FUS treatments

Magnetic resonance-guided FUS (MRgFUS) treatments were performed using the RK100 system (FUS Instruments, Toronto, Canada), consisting of a spherically curved focused transducer driven at 1.68 MHz (75 mm diameter, 60 mm ROC), which generated a focal spot 0.73 mm × 4.5 mm in the lateral and axial planes, respectively^17^. The following sonication parameters for BBB treatment were used: 10 ms bursts, 1 Hz burst repetition frequency, lasting 120 s in total^12,15–17,64^. Acoustic emissions were received by a polyvinylidene difluoride hydrophone located in the center of the transducer. The spatial coordinates of the RK100 were co-registered to that of the 7T MRI scanner (BioSpin 7030; Bruker, Billerica, MA) (Supplementary Fig. S9).

Anesthetized mice were placed supine on a MRI-compatible sled made in-house. The head was coupled to a water-bath with ultrasound gel. T2-weighted images (TR = 2000/TE = 60) were acquired to visualize the brain anatomy for targeting purposes, and T1-weighted images were acquired (TR = 500/TE = 10) pre- and post-sonication to assess BBB permeability (Fig. 1). Four target spots, two per hemisphere, were chosen along the dorsal hippocampus, since it is considered to be equivalent to the posterior hippocampus in humans and important in memory systems affected in AD^65^. Animals were given an intravenous dose of Definity microbubble (MB) contrast agent (Lantheus Medical Imaging, North Billerica, MA), diluted 1:10 (v/v) in saline, at a dose of 0.02 mL/kg, prior to sonication. During sonication, forward and reflected power levels were recorded using a power meter. Acoustic pressures were incrementally increased by 0.025 MPa every burst until subharmonic emissions from microbubbles reached a threshold of 3.5 times the magnitude of background signals, at which point acoustic pressure was automatically reduced by 50% and maintained for the remainder of the sonication duration^48^. After sonication, Gadovist, a gadolinium-based contrast agent (diluted 1:50 in saline (v/v), 0.1 mL/kg of animal mass) was administered intravenously, and a T1-weighted image was acquired to confirm increased BBB permeability.

Control animals received weight-equivalent doses of MBs and gadolinium contrast agent and spent equal time anesthetized in the MR, but were not exposed to FUS.

#### Evaluation of BBB permeability in T1w MRIs

BBB permeability was assessed by finding the relative enhancement of the treatment spots to untreated regions in the brain in post-sonication T1-weighted images. The pixel intensity of a 3 × 3 pixel area was measured for each of the four targeted spots, and the mean intensity value for all four targets was calculated. Relative enhancement was calculated by finding the ratio of this value to the mean intensity of the background, which was an untargeted area in the brain in the same image.

#### Tissue processing for MRgFUS experiments

Animals were anesthetized with intramuscular injections of a ketamine-xylazine cocktail (2:1) and perfused intracardially with ice-cold saline and 4% paraformaldehyde. Brains were removed and immersed in 4% paraformaldehyde at 4 °C overnight, cryoprotected with 30% sucrose immersion and 0.1% sodium azide at 4 °C overnight, embedded in OCT (Sakura Finetek, VWR, PA, USA), and stored at −80 °C. Brains were cryosectioned into 40 μm thick coronal sections, and kept in cryoprotectant (20% glycerin, 30% ethylene glycol, 50% 0.1 M phosphate buffer) at −10 °C.

One in four serial sections were used for immunohistochemical analysis of plaques. Mouse anti-6F3D antibody (1:200, Dako, Glostrup, Denmark) and donkey anti-mouse Alexa Fluor 555 (1:200; Invitrogen, Ontario, Canada) were used to fluorescently label Aβ plaques.

#### Stereological plaque analysis for MRgFUS experiments

Stereological Aβ plaque analysis was conducted using StereoInvestigator software (MBF Bioscience, Vermont, USA). Anatomical boundaries of the dorsal hippocampus were defined by the boundaries of the alveus, third ventricle, and thalamus. The 5× objective was used for contour tracing, and the 20× objective was used for counting plaques. Two stereological probes were used: the optical fractionator probe to count the number of plaques, and the nucleator probe to measure the maximum cross-sectional area of counted plaques. A pilot study was first conducted to determine appropriate stereological parameters for both probes. The following parameters were used for the optical fractionator: counting frame area = 200 × 200 μm, sampling grid area = 500 × 500 μm, optical disector height = 20 μm, guard zone height = 2 μm, and section sampling frame = 1/4. The average coefficient of error (CE Schmitz-Hof) was 0.27 and 0.24 for the estimations of the number of plaques for Tg FUS and Tg CTL mice, respectively. Only plaques that were entirely contained within the section were counted. For each counted plaque, its maximum cross-sectional area was measured by marking its boundaries on four rays that radiated from the center of the plaque.

Brain surface area covered by Aβ plaques, or plaque surface area, per section was determined by finding the product of the average number of plaques (optical fractionator) and the corresponding maximum cross-sectional area (nucleator).

#### Statistical analysis

Statistical analyses were performed using GraphPad Prism (Prism version 7.03 for Windows, GraphPad Software, La Jolla California USA). To evaluate animal mortality, two Fisher’s exact tests with genotype (Tg vs nTg) or treatment (FUS vs CTL) as the nominal variables were used. To evaluate weight fluctuation, a two-way ANOVA with ‘Group’ and ‘Treatment Number’ as the two independent variables was used. For comparison of post-sonication T1-weighted contrast enhanced images, an unpaired *t*-test was used. For comparison of plaque number, area, and surface area, an unpaired two-tailed *t*-test was used to evaluate Tg FUS and Tg CTL groups.

## Electronic supplementary material

Supplementary Figures

Supplementary Materials

## Data Availability

The datasets generated during this study are available from Charissa Poon upon reasonable request.
