# Supplementary material for: Time course of focused ultrasound effects on β-amyloid plaque pathology in the TgCRND8 mouse model of Alzheimer’s disease
Source: Sci Rep. 2018 Sep 19;8:14061. doi: 10.1038/s41598-018-32250-3 (PMC6145880; doi:10.1038/s41598-018-32250-3)
Supplement: Supplementary file 1 — Supplementary Figures [file 41598_2018_32250_MOESM1_ESM.pdf]

Supplementary Figures: Time course of focused ultrasound effects on β-amyloid pathology in the TgCRND8 mouse model of Alzheimer's disease

Charissa T Poon[1,2*], Kairavi Shah[2], Chiungting Lin[2], Ryan Tse[2], Kate K Kim[2], Skyler Mooney[2], Isabelle Aubert[4,5], Bojana Stefanovic[2,3], and Kullervo Hynynen[1,2,3]

[1] Physical Sciences Platform, Sunnybrook Research Institute, Toronto, Ontario, Canada

[2] Institute of Biomaterials and Biomedical Engineering, University of Toronto, Toronto, Ontario, Canada

[3] Department of Medical Biophysics, University of Toronto, Toronto, Ontario, Canada

[4] Biological Sciences Platform, Sunnybrook Research Institute, Toronto, Ontario, Canada

[5] Department of Laboratory Medicine and Pathobiology, University of Toronto, Toronto, Ontario, Canada

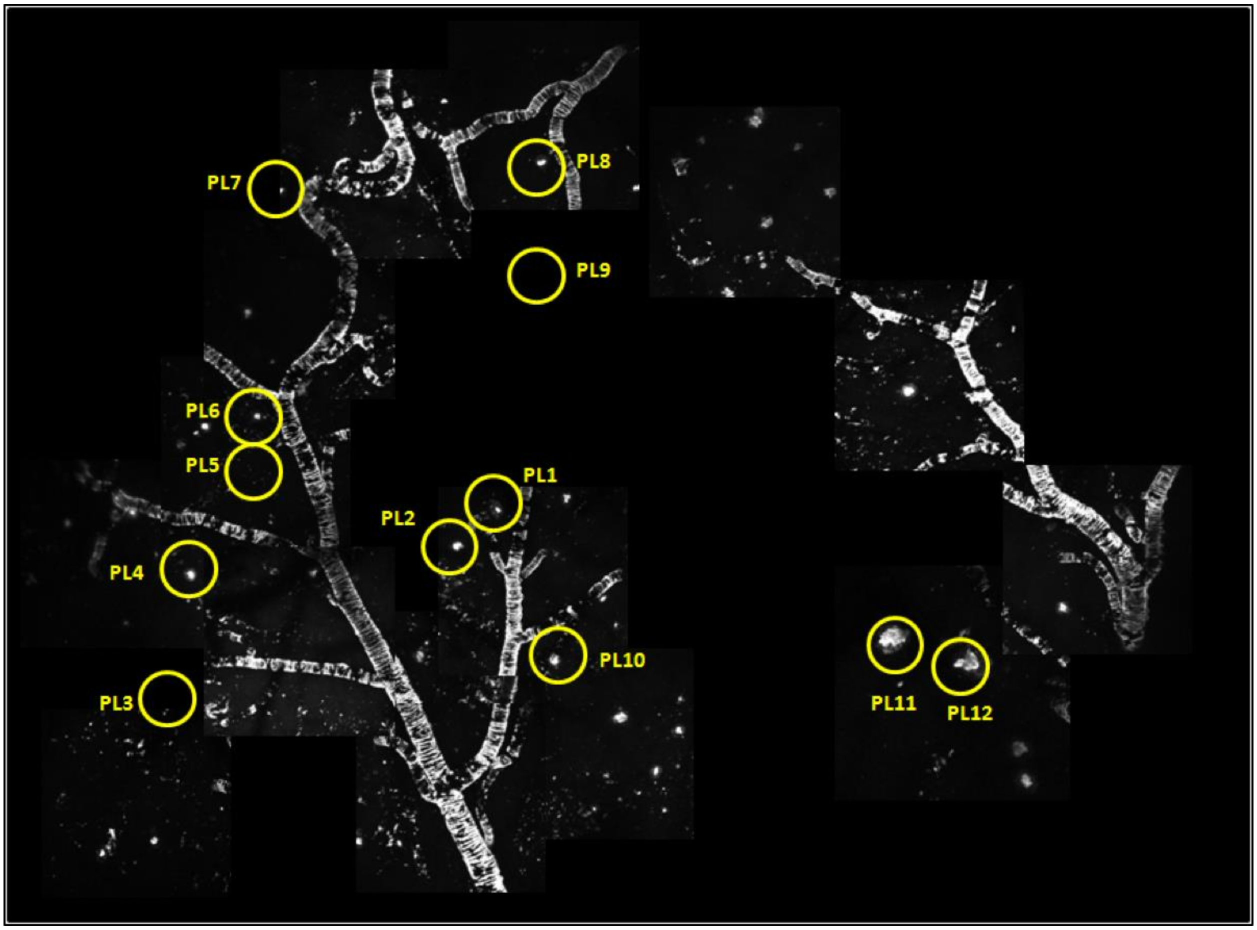

Supplementary Figure S1. Aβ plaque map.
Plaque maps were created by stitching together shallow depth stacks of amyloid-covered blood vessels. An average of 10 plaques were selected for each animal. The location of each plaque to blood vessels was marked to facilitate relocation in the three subsequent weeks of imaging.

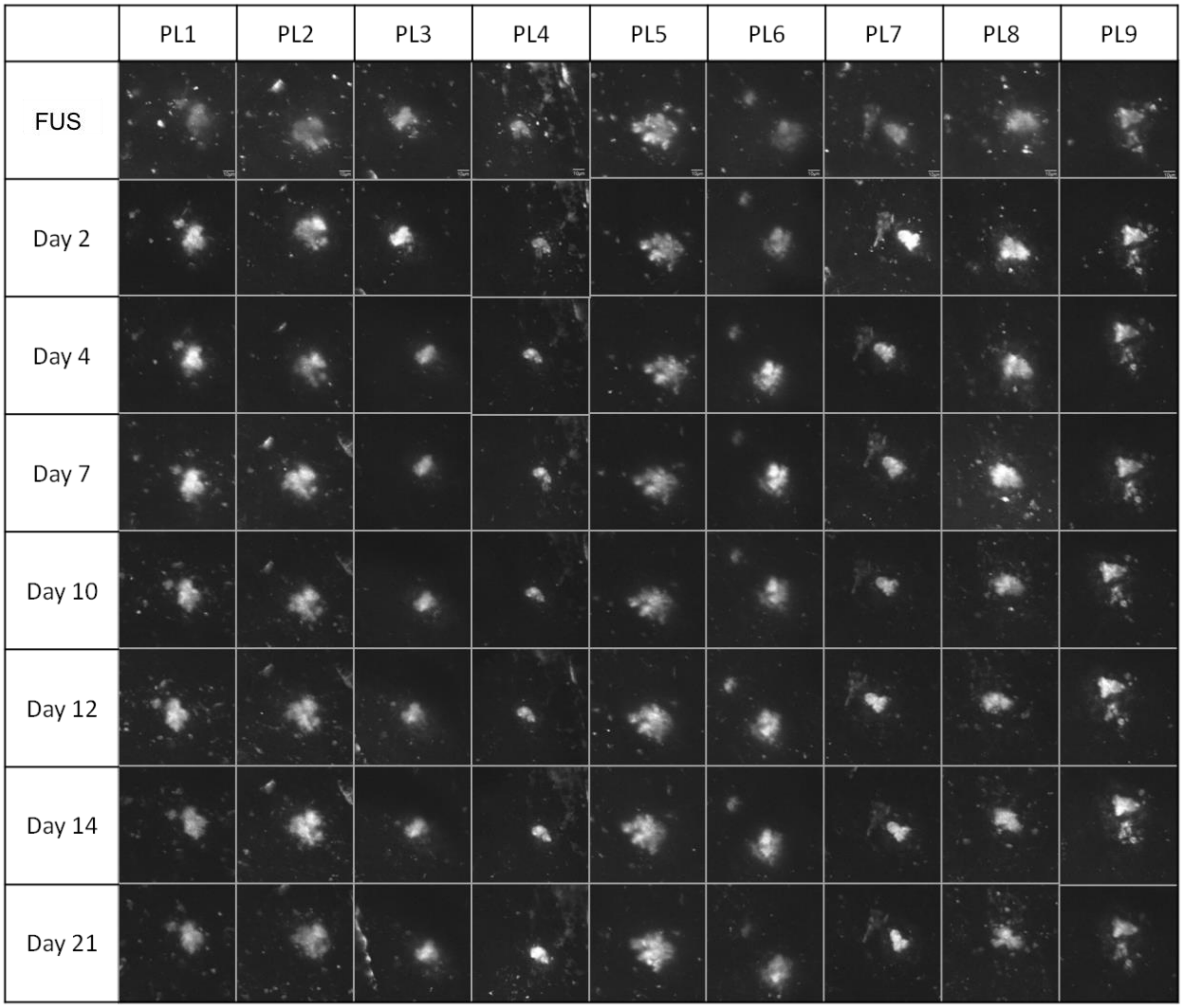

Supplementary Figure S2. Plaque chart.
Maximum projection images of the raw XYZ image stacks of all the plaques collected for one animal are shown. Each column represents a different plaque, and each row represents a different imaging day.

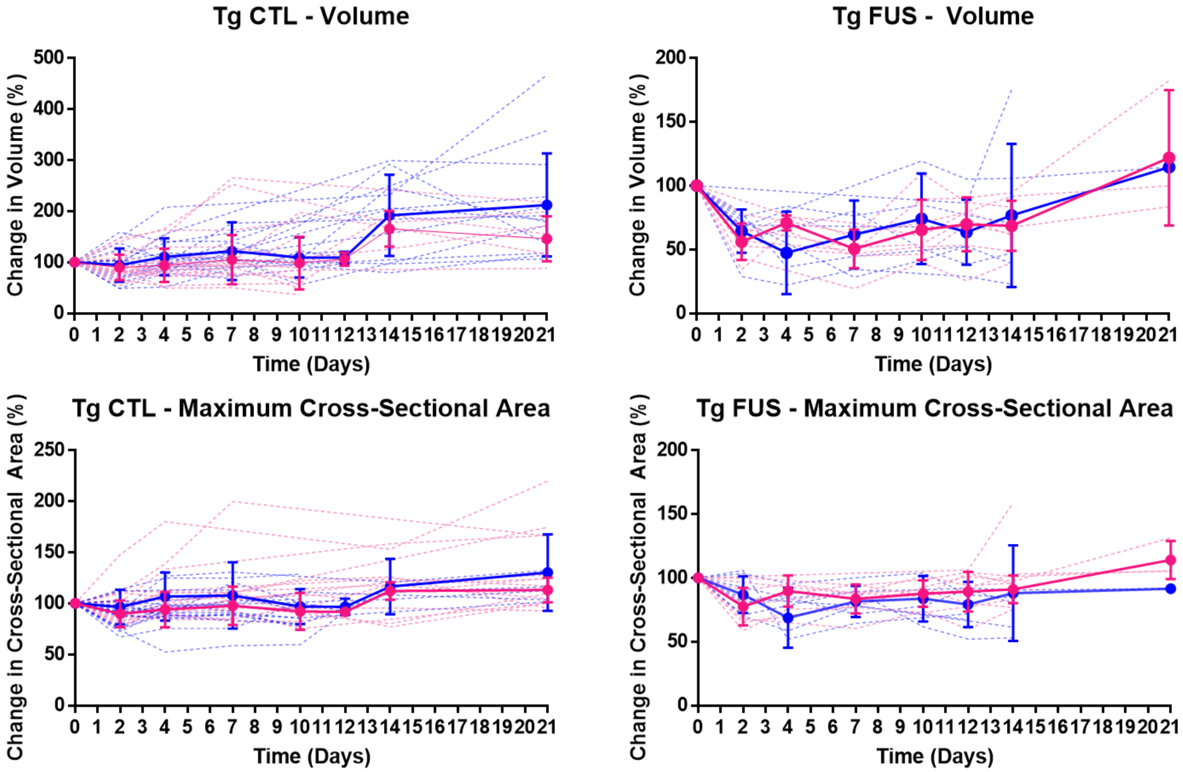

Supplementary Figure S3. Changes in volume and cross-sectional area of larger and smaller plaques in Tg CTL (left) and Tg FUS (right) animals. The same data as that in Figure 3 is shown, but binned by size into larger (magenta) and smaller (blue) plaques based on the median of raw volume values measured on day 0. A t-test revealed that there is no difference in changes in volume or cross-sectional area between larger and smaller plaques (Tg CTL: $p = 0.3$ for volume, $p = 0.2$ for maximum cross-sectional area, Tg FUS: $p = 0.8$ for volume, $p = 0.5$ for maximum cross-sectional area,). Solid lines show mean ± SD values, dotted lines show individual plaques.

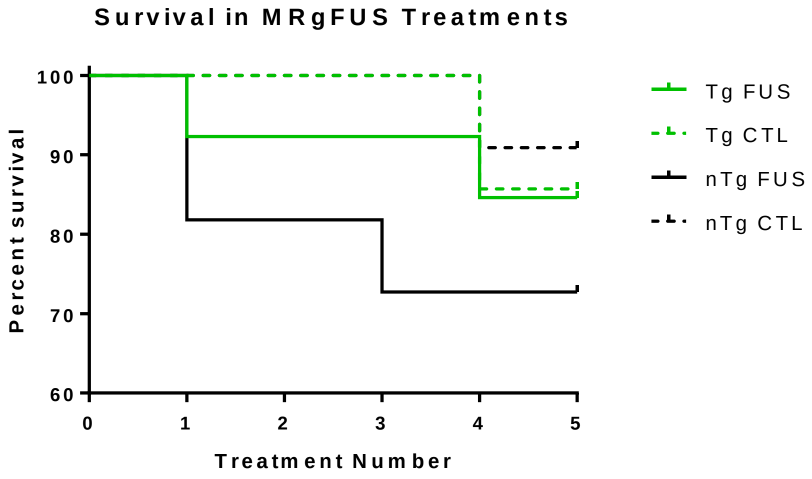

Treatment	Tg FUS	Tg CTL	nTg FUS	nTg CTL
Pre-FUS	13	7	11	11
1	13	7	11	11
2	13	7	9	11
3	12	7	9	11
4	11	6	8	10
5	11	6	8	10
Number of deaths	2	1	3	1

	alive	dead	total
Tg	17	3	20
nTg	18	4	22

	alive	dead	total
FUS	21	5	26
CTL	16	2	18

Supplementary Figure S4. Survival curve in MRgFUS study. No difference in mortality of Tg and nTg animals in 10-week MRgFUS study.

Fisher's exact test shows that there is no significant difference in mortality between genotypes (Tg vs nTg, $p > 0.99$) or treatment (FUS vs CTL, $p = 0.68$).

Animal ID	Group	Number of FUS treatments delivered
85833	Tg FUS	5
86015	Tg FUS	4
86036	Tg FUS	4
86056	Tg FUS	4
86071	Tg FUS	3
85815	nTg FUS	4
85838	nTg FUS	5
86074	nTg FUS	4
86022	nTg FUS	5
86029	nTg FUS	5
86015	nTg FUS	5
86067	nTg FUS	5

Supplementary Table S5. Number of FUS treatments that Tg FUS and nTg FUS mice received in the MRgFUS study.

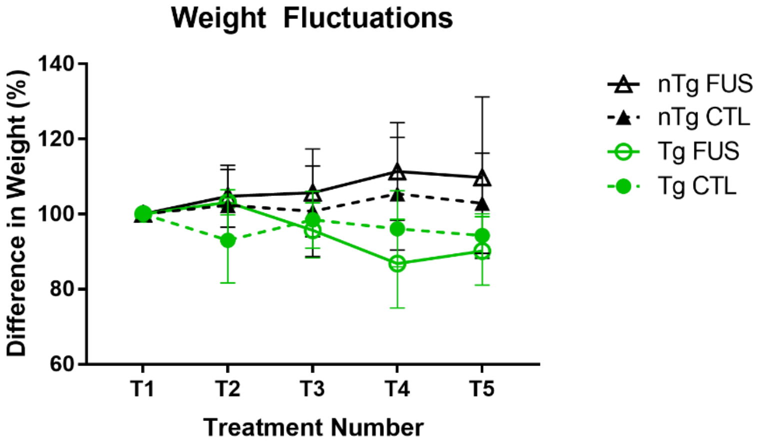

	nTg FUS	nTg CTL	Tg CTL	Tg FUS
T1	100 ± 0	100 ± 0	100 ± 0	100 ± 0
T2	105 ± 8	102 ± 10	93 ± 11	103 ± 3
T3	106 ± 12	104 ± 12	98 ± 8	96 ± 7
T4	111 ± 13	105 ±15	96 ± 10	87 ± 12
T5	110 ± 22	103 ± 13	94 ± 6	90 ± 9
n	7	17	5	5

Supplementary Figure S6. Weight fluctuations during MRgFUS study.
Subjects' weights fluctuated within 13% of their starting weight during the 5 FUS treatments. Differences in weight was significantly affected by genotype (p = 0.0002), but not treatment number ($p > 0.9$). Data points show the normalized mean ± SD of each treatment group on a given treatment day.

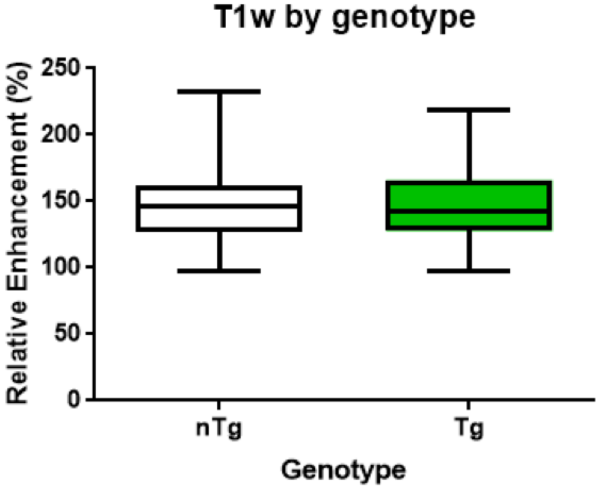

	nTg	Tg
Relative enhancement values	153 ± 24	146 ± 23
n	7	5

Supplementary Figure S7. Comparisons in BBB permeability between Tg and nTg groups. Increases in BBB permeability were evaluated by comparing the FUS-targeted regions with untargeted regions in the brain (baseline), in contrast-enhanced T1-weighted MRIs (nTg n = 7, Tg n = 5; mean ± SD; p = 0.9, unpaired t-test). Box-and-whiskers plots show mean and range of each group.

	**Time course of 1 FUS treatment**	**Effects of repeated biweekly treatments**
**Imaging modality**	*In vivo* two photon fluorescence microscopy	Treatments guided by MRI<br>Aβ analyzed using IHC, stereology
**Aβ-specific labelling**	Methoxy X-04	6F3D antibody
**Brain volume targeted**	Up to 1 mm below the brain surface (limited by transducer design)	Bilateral hippocampi
**Attributes analyzed**	Aβ plaque volume and maximum cross-sectional area	Aβ number, maximum cross-sectional area, and total surface area of brain section covered by Aβ

Supplementary Table S8. Comparison of the two-photon fluorescence microscopy and MR-guided FUS studies.

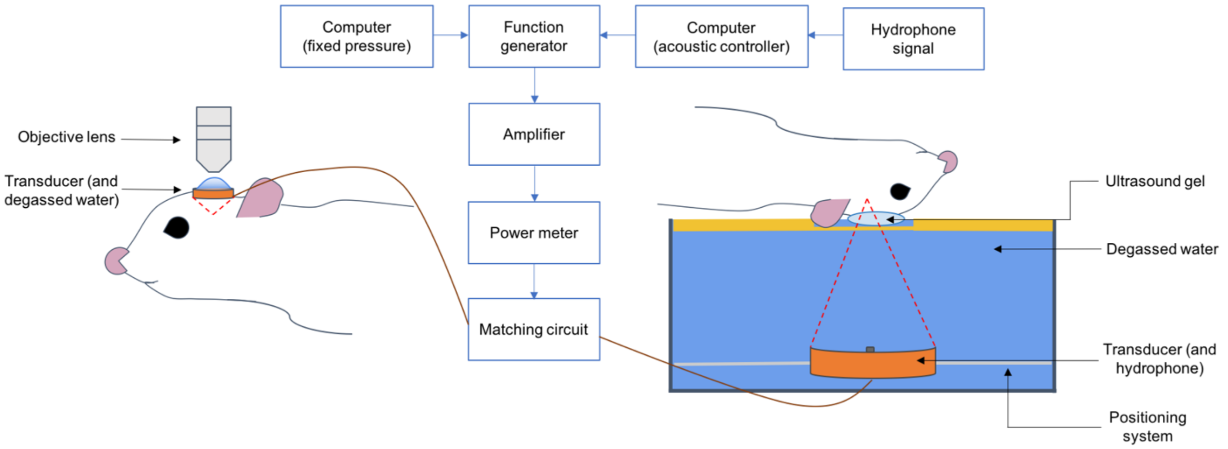

Supplementary Figure S9. Two photon fluorescence microscopy FUS system (left) and MRgFUS system (right). *Left*: In the two photon fluorescence microscopy FUS system, the ring transducer was positioned above the cranial window. A drop of degassed water was placed in the center of the transducer to accommodate the water-immersion objective lenses. Two photon fluorescence microscopy FUS experiments were operated at fixed pressures. *Right:* In the MRgFUS system, a spherically curved transducer and polyvinylidene difluoride hydrophone were positioned in a water tank below the subject's head. Acoustic signals from microbubble activity were measured using the hydrophone, and used to control acoustic pressures during the duration of sonication. Dotted red lines indicate the approximate focus of the transducers (not to scale).

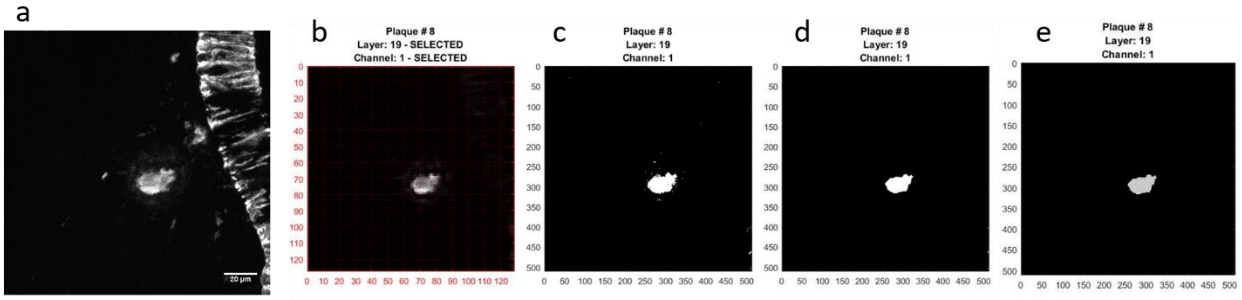

Supplementary Figure S10. Image processing workflow of two-photon fluorescence microscopy image stacks of plaques. A maximum projection image of the raw image stack of a plaque is shown in (a). A nearby CAA-covered blood vessel can also be observed. XYZ image stacks collected with the two-photon microscope underwent the following image processing steps: importing image stack, identifying ROI (b; only step requiring user input), thresholding (c), defragmenting (d), computing volume (e). Scale bar = 20 μm.

	Volume	Cross-Sectional Area
day 0 (FUS)	100 ± 0	100 ± 0
day 2	92 ± 29	93 ± 15
day 4	103 ± 35	101 ± 22
day 7	112 ± 52	102 ± 25
day 10	102 ± 47	94 ± 18
day 12	108 ± 11	95 ± 6
day 14	186 ± 71	116 ± 24
day 21	195 ± 93	126 ± 33

Supplementary Table S11. Data tables for *Figures 3a, b*: Tg CTL animals demonstrated a linear increase in plaque growth over three weeks.

	Volume	Cross-Sectional Area
day 0 (FUS)	100 ± 0	100 ± 0
day 2	62 ± 16	85 ± 15
day 4	59 ± 22	79 ± 19
day 7	54 ± 21	84 ± 14
day 10	65 ± 27	87 ± 13
day 12	63 ± 24	84 ± 17
day 14	67 ± 38	90 ± 23
day 21	96 ± 51	102 ± 20

Supplementary Table S12. Data tables for *Figures 3c, d*: Tg FUS animals demonstrated a rapid decrease in plaque volume by day 2 after FUS treatment.

**Number**		**Maximum Cross-Sectional Area**		**Surface Area**	
**CTL**	**FUS**	**CTL**	**FUS**	**CTL**	**FUS**
466	274	403	261	20500	7790
406	313	350	359	15100	11600
423	275	303	293	12400	8340
408	399	321	279	14100	9070
372	250	350	362	14600	9520
**420 ± 20**	**300 ± 30**	**350 ± 20**	**310 ± 20**	**15400 ± 1360**	**9270 ± 660**
Difference between means: 110 ± 30, $p \leq 0.01$		Difference between means: 40 ± 30, $p = 0.24$		Difference between means: 6100 ± 1500, $p \leq 0.01$	

Supplementary Table S13. Data tables for *Figure 4*: Boxplots showing comparisons of plaque number and surface area between Tg FUS and Tg CTL animals.
