# Supplementary material for: Time course of focused ultrasound effects on β-amyloid plaque pathology in the TgCRND8 mouse model of Alzheimer’s disease
Source: Sci Rep. 2018 Sep 19;8:14061. doi: 10.1038/s41598-018-32250-3 (PMC6145880; doi:10.1038/s41598-018-32250-3)
Supplement: Supplementary file 2 — Supplementary Materials [file 41598_2018_32250_MOESM2_ESM.pdf]

Supplementary Materials: Time course of focused ultrasound effects on β-amyloid plaque pathology in the TgCRND8 mouse model of Alzheimer's disease

Charissa T Poon[1,2*], Kairavi Shah[1], Chiungting Lin[1], Ryan Tse[1], Kate K Kim[1], Skyler Mooney[1], Isabelle Aubert[4,5], Bojana Stefanovic[1,3], and Kullervo Hynynen[1,2,3]

[1] Physical Sciences Platform, Sunnybrook Research Institute, Toronto, Ontario, Canada

[2] Institute of Biomaterials and Biomedical Engineering, University of Toronto, Toronto, Ontario, Canada

[3] Department of Medical Biophysics, University of Toronto, Toronto, Ontario, Canada

[4] Biological Sciences Platform, Sunnybrook Research Institute, Toronto, Ontario, Canada

[5] Department of Laboratory Medicine and Pathobiology, University of Toronto, Toronto, Ontario, Canad

This document describes the steps taken to develop a correction factor for the effect of laser power on measured plaque volume and maximum cross-sectional area.

Power measurements of the 780 nm excitation laser are shown below. The relationship between laser power (LP) percentage and output power was found to be: power (mW) = 2.058*LP

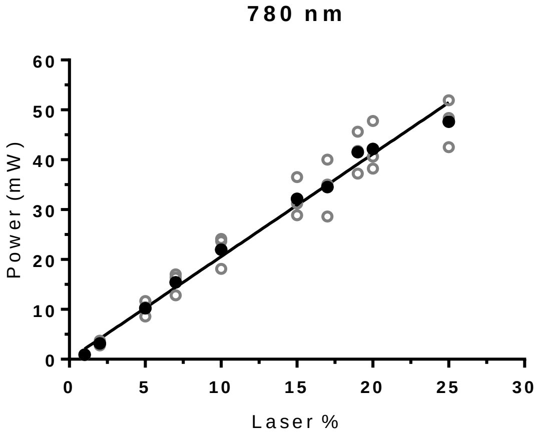

Laser Power (%)	Power (mean ± SEM, mW)
1	0.9 ± 0.1
2	3.2 ± 0.3
5	10.2 ± 0.9
7	15.4 ± 1.3
10	22.0 ± 1.9
15	32.2 ± 2.3
17	34.5 ± 3.3
19	41.5 ± 2.4
20	42.2 ± 2.9
25	47.6 ± 2.7

Supplementary Figure SM1: Laser power (%) and output power (mW) using 780nm excitation laser.

Using these measurements, we graphed all the laser powers used for each plaque across all imaging days: (Tg SON - blue, Tg CTL - red). Small variations in laser power to acquire optimal image quality are typical in microscopy.

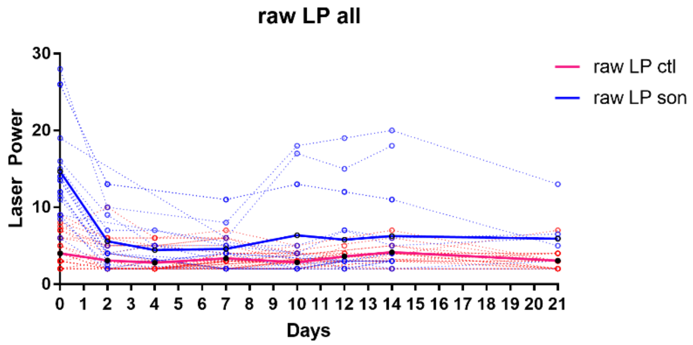

	SON		CTL	
Time (Days)	Mean ± SD (mW)	*n*	Mean ± SD (mW)	*n*
0	27.4 ± 15.1	18	8.3 ± 4.0	36
2	11.5 ± 7.6	16	6.3 ± 3.7	36
4	9.1 ± 3.1	7	5.8 ± 2.7	35
7	9.4 ± 6.0	17	6.9 ± 3.3	27
10	13.1 ± 12.4	14	5.8 ± 1.9	18
12	11.9 ± 10.7	18	7.4 ± 2.3	5
14	12.9 ± 11.8	16	8.5 ± 2.8	13
21	12.2 ± 8.0	6	6.3 ± 2.5	19

Supplementary Figure SM2: Laser powers (mW) used for each plaque analyzed across all imaging days.

Laser powers used on day 0 were found to be consistently higher than those used on subsequent imaging days in Tg FUS-treated animals. This is due to the leakage of the fluorescent dextran into the extravascular space during FUS-induced BBB treatment on day 0. Since image stacks of plaques were acquired after FUS treatment, crosstalk from the Texas Red dextran increased background fluorescence in the methoxy-X04 channel, thereby requiring higher laser power to achieve adequate SNR. In Tg CTL animals, laser powers used on day 0 were consistent with all other imaging days.

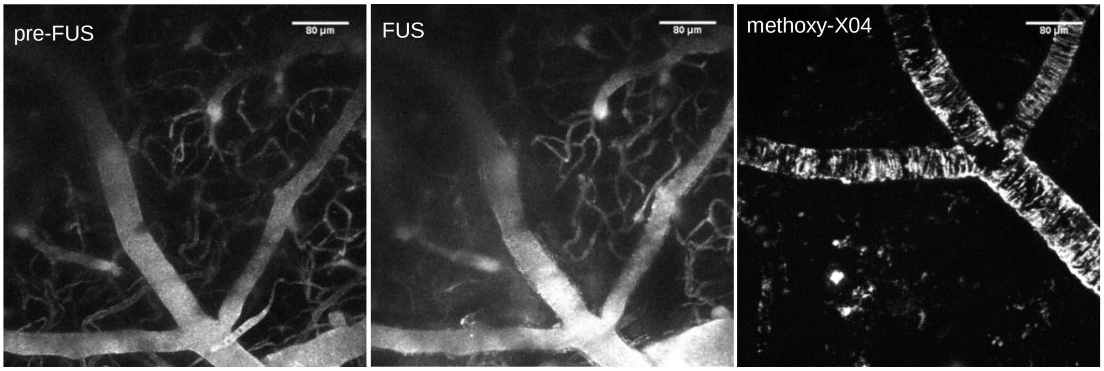

Supplementary Figure SM3: Leakage of fluorescent dextran into the extravascular space during FUS exposure (left, middle). Aβ plaques and CAA are observed in the methoxy-X04 channel (right).

FUS treatment caused leakage of the fluorescent dextran into the extravascular space, shown in the middle panel. The effect of extravascular dextran on the SNR in the methoxy-X04 channel was not always evident (right panel). However, when comparing plaques on different imaging days at a higher zoom, the lower SNR on day 0 from FUS-induced dextran leakage was clear.

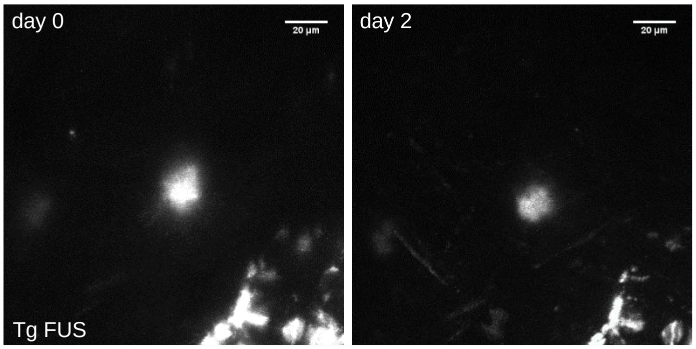

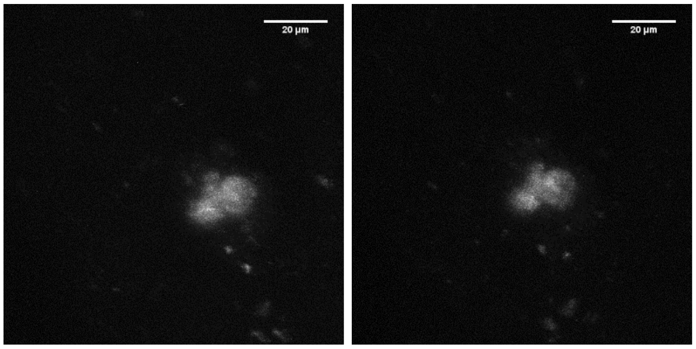

Supplementary Figure SM4: Difference in SNR is clear between day 0 and day 7 in Tg FUS (top) but not Tg CTL (bottom) animals.

Below, we show the normalized laser powers used on each imaging day (black), and the corresponding normalized means of volume (red) and cross-sectional area values (green) obtained from our image analysis algorithm. Unfortunately, the higher laser powers used on day 0 corresponded with the higher plaque volume and maximum cross-sectional area measurements obtained on day 0. To parse out the effect of laser power on plaque size on day 0, the following steps were taken to find a correction factor for each plaque:

1. Find the difference between the laser powers used on day 0 and the average of laser powers used on all other imaging days.
2. Find the relationship between laser power used, and plaque volume and maximum cross-sectional area measurements.
3. Use the value from (1) in the volume and maximum cross-sectional area equations from (2), to get the corrected day 0 plaque volume and maximum cross-sectional area measurements.

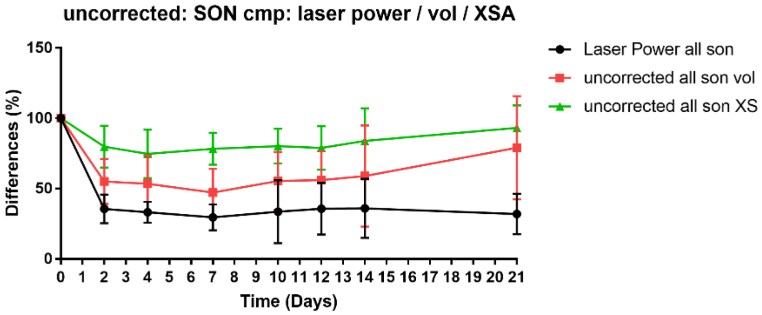

Supplementary Figure SM5: Laser powers, and plaque volume and maximum cross-sectional area measurements of Tg FUS animals across all imaging days.

The find the relationship between laser power and plaque volume and maximum cross-sectional area, multiple image stacks of plaques (*n* = 7) was acquired at different laser powers, and the volume and maximum cross-sectional area was analyzed at each laser power. Linear regressions revealed a linear relationship between measured plaque volume (volume = 2.5*LP + 87) and cross-sectional area (XSA = 1.1*LP + 95).

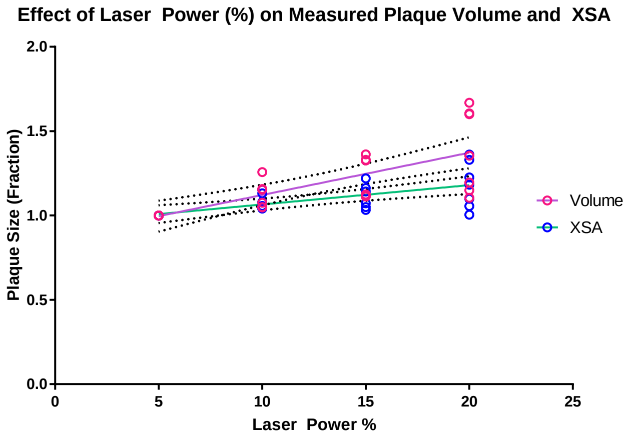

Supplementary Figure SM6: Measured plaque volume and maximum-cross sectional area increase linearly with laser power.

Using these two relationships, we then found the corrected volume and maximum cross-sectional area measurements by substituting the difference in LP used on day 0 and the average of LPs used on all other imaging days for each plaque. LPs used on all other imaging days were relatively consistent. In addition, as previously mentioned, some variation in LP is typical to acquire optimal SNR in microscopy.

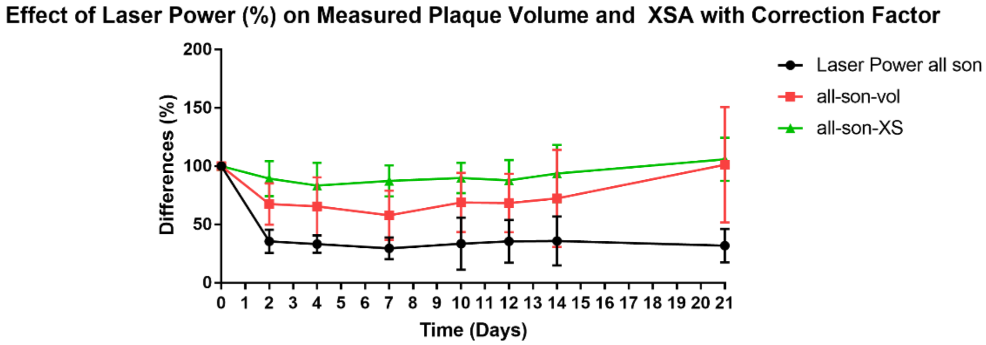

Supplementary Figure SM7: Laser powers, and volume and maximum cross-sectional area measurements of Tg FUS animals across all imaging days.

There remains a decrease in measured plaque volume and maximum cross-sectional area from day 0 to day 2; we believe this reflects the effects of FUS on plaque size.

Linear regressions of plaque volume and maximum cross-sectional area from day 2 to day 21 reveal growth rates of 1.7 ± 0.5% per day, 95% CI slope = 0.4 to 3.1, Sy.x = 8.5 for volume, and 1.0 ± 0.3% per day, 95% CI slope = 0.3 to 1.6, Sy.x = 4.1 for maximum cross-sectional area. Notably, the growth rate of plaque volume is greater in Tg CTL (3.6 ± 0.8%) compared to Tg FUS animals.

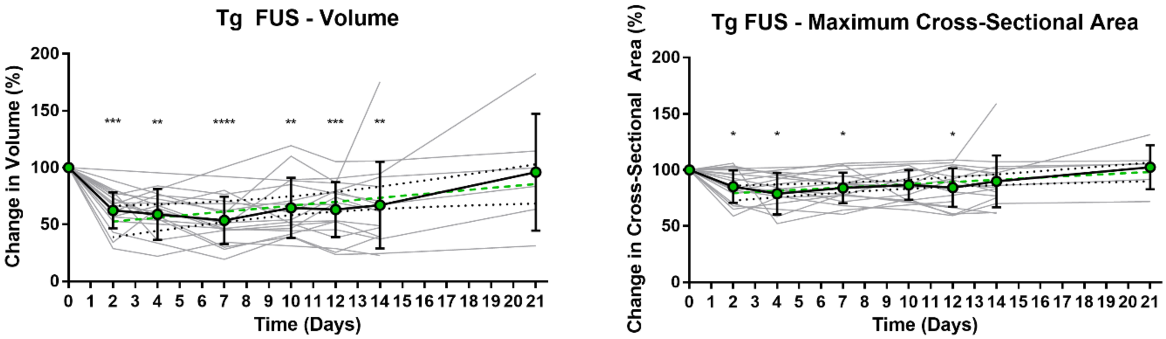

Supplementary Figure SM8: Plaque volumes and maximum cross-sectional areas (as in Fig. 3*) with linear regressions (green, with 95% CI) from day 2 to day 21 in Tg FUS animals.*
